# Ecology drives the degree of convergence in the gene expression of extremophile fishes

**DOI:** 10.1098/rspb.2026.1416

**Published:** 2026-10-21

**Authors:** Michael Tobler, Ryan Greenway, Joanna L. Kelley

**Affiliations:** ^1^ Department of Biology and Whitney R. Harris World Ecology Center, University of Missouri-St. Louis, St. Louis, MO, USA; ^2^ WildCare Institute, Saint Louis Zoo, St. Louis, MO, USA; ^3^ Max Planck Institute of Animal Behavior, Konstanz, Germany; ^4^ Ecology and Evolutionary Biology Department, University of California Santa Cruz, Santa Cruz, CA, USA

**Keywords:** comparative transcriptomics, convergent evolution, hydrogen sulfide, phylogenetic comparative analyses, Poeciliidae

## Abstract

Convergent evolution is common and a testament to the repeatability of evolution. Still, non-convergence is also widespread, and a major question is whether idiosyncratic, lineage-specific changes are reflective of chance events inherent to evolutionary processes, or whether they are influenced by deterministic genetic or ecological factors. To address this question, we quantified the degree of convergence in gene expression across lineages of livebearing fishes (Poeciliidae) that span 40 million years of divergence and have colonized toxic, hydrogen-sulfide-rich springs. We asked whether the degree of convergence across lineage pairs was related to their phylogenetic relatedness and the ecological similarity of their habitats. Using phylogenetic comparative analyses, we showed that the degree of convergence in gene expression was variable across lineage pairs. While closely related lineages did not exhibit higher degrees of convergence than distantly related ones, we uncovered a strong relationship between degree of convergence and the ecological similarity among lineages. These results suggest that variation in environmental conditions that is often overlooked in simplified descriptions of habitat differences can play an important role in shaping adaptive evolution. Our study highlights the importance of multivariate approaches that capture the complexities of selective regimes and organismal responses when assessing the repeatability of evolution.

## 1. Introduction

A core question in biology is whether evolution repeats itself and, if so, whether we can predict the circumstances under which repeatable evolutionary change happens [1]. The question of evolutionary repeatability is epitomized by the contrasting perspectives of two giants in the field. On the one side, Stephen Jay Gould was adamant about the role of contingency in evolution, famously concluding that the ‘tape of life’ would never repeat itself owing to chance events inherent to the evolutionary process [2]. On the other side, Simon Conway Morris, a strong proponent of evolutionary determinism, likened evolution to a drop of water pulled by gravity along a predictable path of least resistance, inevitably ending up in the ocean. Drawing upon much of the same evidence as Gould, he argued that evolution does repeat itself, because there are a finite number of engineering solutions to the problems posed by a finite set of niches available to life on earth [3].

Past research has unearthed much evidence for both contingency and determinism in evolution. The importance of random mutation and genetic drift in evolutionary change is just as undeniable [4] as the prevalence of convergent evolution [5–7], where independent lineages evolve similar adaptive solutions to shared ecological problems. Hence, the question is not so much whether chance events happen, or whether evolution can repeat itself, but instead whether we can predict the circumstances under which determinism prevails over contingency, recognizing that similar phenotypes may arise through both adaptive and non-adaptive processes [7,8]. At the root of this problem are confirmation biases inherent to the detection of deterministic and contingent evolutionary outcomes. It is relatively easy to document convergence [9–11]. However, a lack of convergence is not necessarily evidence for contingency; failure to understand the origins of lineage-specific idiosyncrasies does not exclude deterministic explanations in favour of invoking contingency. In fact, unique, lineage-specific evolutionary outcomes in response to apparently similar selective regimes can arise through a multitude of genetic, functional and ecological mechanisms [12]. For example, differences in genetic architecture can bias responses to selection [13], functional redundancy can cause equivalent fitness by alternative means [14,15], and ecological differences among lineages—even within broadly similar habitat types—can select for different phenotypic optima [16]. Still, attempts to explain the degree of convergent and lineage-specific (i.e. nonconvergent) evolutionary patterns remain scarce.

We leveraged a unique natural experiment to test whether we can explain variation in the degree of convergent evolution in response to a strong and clearly defined source of selection. Throughout the Neotropics, fishes of the family Poeciliidae have colonized springs rich in hydrogen sulfide (H_2_S) [17], a potent respiratory toxicant lethal to most metazoans in micromolar concentrations [18]. Sulfide spring lineages span over 40 million years of evolutionary divergence, facilitating comparisons within species, among species, and across genera (figure 1A). Past research has documented clear signatures of convergent evolution among sulfide spring lineages at multiple biological levels [17,19]. For example, mitochondrial genomes exhibit convergent amino acid substitutions in cytochrome c oxidase (COX), a key toxicity target, which reduce sensitivity to H_2_S [20–22]. Sulfide spring lineages also show convergent regulatory shifts, including upregulation of H_2_S detoxification pathways [21,23]. Additionally, convergence is evident in physiological function (increased tolerance to both H_2_S and hypoxia), morphology (traits associated with ventilation and foraging) and reproductive life histories (shifts from *r*- towards *K*-selected strategies) [24–29]. Despite the evidence for convergence across levels of organismal organization and across a wide gradient of phylogenetic relationships, past studies have also found evidence of lineage-specific evolutionary change, giving rise to substantial variation in the degree of convergence between pairs of sulfide spring lineages [22,30]. However, these studies have typically assessed convergence across all H_2_S-adapted lineages, rather than explicitly quantifying how much convergence occurs between specific pairs of lineages.

**Figure 1:**
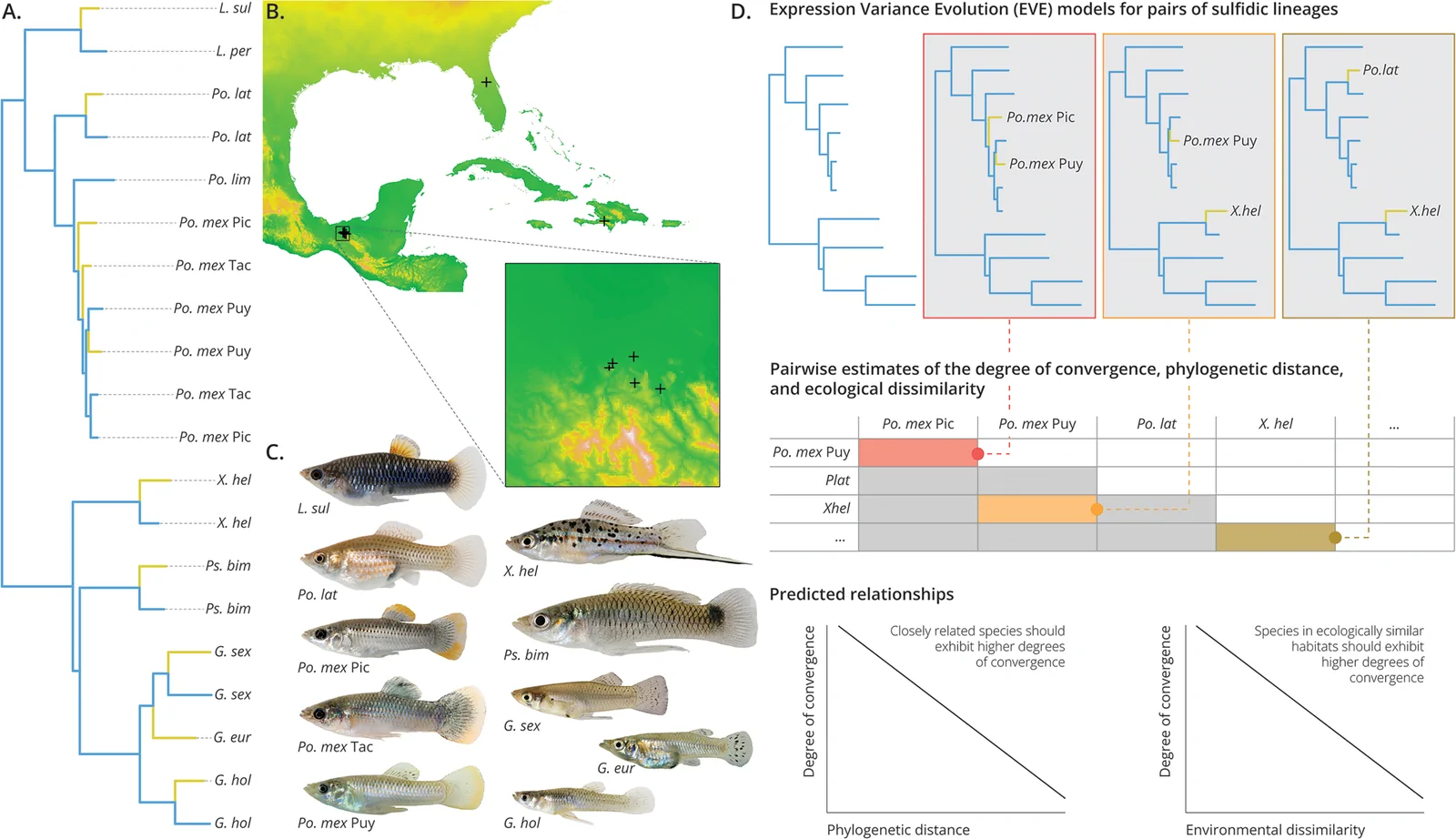
A) Phylogeny of lineages included in this study. Yellow edges indicate lineages that have colonized sulfide springs. (B) Map of the locations of different sulfide springs. Samples were collected from Florida (one spring), the Dominican Republic (one spring) and Mexico (five springs; region enlarged in inset). (C) Representative specimens of the sulfide spring lineages: *Limia sulphurophila* (*L. sul*); *Poecilia latipinna* (*Po. lat*); *Po. mexicana* (*Po. mex*) from the Ríos Pichucalco (Pic), Tacotalpa (Tac) and Puyacatengo (Puy); *Xiphophorus hellerii* (*X. hel*); *Pseudoxiphophorus bimaculatus* (*Ps. bim*); *Gambusia sexradiata* (*G. sex*); *G. eurystoma* (*G. eur*); and *G. holbrooki* (*G. hol*). (D) Outline of the analytical framework used in this study. A phylogeny of all nonsulfidic lineages served as a backbone for all analyses, accounting for gene expression variation that arose as a consequence of the diversification of lineages. To identify convergent shifts in gene expression upon the colonization of sulfide springs, sulfidic lineages were added to the backbone phylogeny in a pairwise fashion (as exemplified by the red, orange and brown outlined phylogenies) to run expression variance and evolution (EVE) models. The output of EVE models provided quantitative measures of the degree of convergence between pairs of sulfide spring lineages, which were complemented by estimates of phylogenetic divergence and ecological dissimilarity. The degree of convergence between pairs of sulfide spring populations was predicted to be negatively correlated both with their phylogenetic distance and the ecological dissimilarity of their habitats.

Here, we build on this framework by shifting the focus from whether convergence occurs across all sulfide spring lineages to how much convergence occurs between specific lineage pairs. This pairwise perspective reveals substantial variation in the degree of convergence and enables us to ask what factors explain that variation. Specifically, we tested whether we could explain variation in the degree of convergence among 10 sulfide spring populations of the family Poeciliidae, including lineages in the genera *Limia, Poecilia, Xiphophorus, Pseudoxiphophorus* and *Gambusia* from three different biogeographic regions (figure 1A–C). We used phylogenetic comparative methods to analyse patterns of gene expression and asked whether convergence between pairs of sulfide spring populations was dependent on their phylogenetic relationship or the ecological similarity of their habitats. We predicted that more closely related species would exhibit higher degrees of convergence [31,32], because shared genomic architectures would determine similar responses to selection (figure 1D). In addition, we predicted that species exposed to more similar ecological conditions would exhibit higher degrees of convergence [16,33], because environmental variables that covary with the presence of H_2_S would determine the trajectories of evolutionary change (figure 1D).

## 2. Material and methods

### (a) Sampling

Transcriptomes were reanalysed from a previous study that conducted RNA sequencing (RNAseq) on gill tissues [21]. Gills were studied because they are in direct contact with environmental H_2_S [18], are the site of many physiological processes involved in the maintenance of homeostasis [34] and exhibit strong and heritable transcriptional responses to H_2_S exposure [23,35]. Gill tissues for transcriptome sequencing were collected from 10 lineages of poeciliid fishes that independently colonized sulfide springs in the United States, Mexico and the Dominican Republic (supplementary material, table S1). For comparison, we also included 10 closely related lineages from adjacent nonsulfidic habitats (supplementary material, table S1). The sample size was *n* = 6 for all lineages, except for the sulfidic *Poecilia mexicana* populations from the Tacotalpa (Tac) and Puyacatengo (Puy) river basins (*n* = 5; supplementary material, table S1), for a total of *N* = 118. All fish were collected by seine, and the gills of adult females were extracted from both sides of the body immediately after capture. All tissues were preserved in RNAlater (Ambion Inc., TX, USA) and stored at −20°C upon return to the laboratory.

### (b) Quantifying gene expression

For transcriptome sequencing, we extracted total RNA from pulverized tissue using the NucleoSpin RNA kit (Machery-Nagel, Düren, Germany). We then isolated mRNA with the NEBNext Poly(A) mRNA Magnetic Isolation Module (New England Biolabs, Inc., Ipswich, MA, USA) and constructed cDNA libraries with the NEBNext Ultra Directional RNA Library Prep Kit for Illumina (New England Biolabs, Inc., Ipswich, MA, USA). All cDNA libraries were individually barcoded, quantified using Qubit (Thermo Fisher Scientific, MA, USA) and an Agilent 2100 Bioanalyzer High Sensitivity DNA chip (Agilent, CA, USA), and pooled in sets of 11–12 samples based on nanomolar concentrations. All libraries were sequenced on an Illumina HiSeq 2500 with paired-end 101 base-pair (bp) reads at the Washington State University Spokane Genomics Core. All raw sequencing data are available at the National Center for Biotechnology Information (NCBI) under BioProject accession numbers PRJNA473350 and PRJNA608180.

Raw reads from Illumina sequencing were sorted by barcode, and we used *Trimgalore!* v. 0.4.0 [36] to remove adapters and low-quality bases. Trimmed reads were then mapped to the *Po. mexicana* reference genome (RefSeq accession number: GCF_001443325.1 [37]) with an appended mitochondrial genome (GenBank accession number: KC992998.1) using *BWA-MEM* [38]. Previous analyses using an alternative reference genome (*Xiphophorus hellerii*) indicated that reference genome choice had little effect on downstream results, yielding largely consistent patterns of differential expression despite variation in phylogenetic distance to the reference genome [21]. Finally, we generated a gene expression matrix (in fragments per kilobase of transcript per million mapped reads, FPKM) for all individuals and the 31 804 genes in the reference genome using *cufflinks* v. 2.2.1 [39]. Genes were filtered to retain those with FPKM values greater than 2 in at least 79 (67%) samples, leaving 11 390 genes for downstream analyses. Genes were annotated by comparing them to the entries in the human SWISSPROT database (critical *E*-value 0.001; access date 15 April 2017) using BLASTx and retaining the top BLAST hit for each gene based on the top high-scoring segment pair. Genes were annotated by comparison to the human SwissProt database because the H_2_S detoxification, sulfur metabolism and mitochondrial pathways central to adaptation to sulfide springs are highly conserved across vertebrates and more comprehensively annotated in the human reference proteome than in currently available teleost resources.

### (c) Phylogenetic analysis

The phylogenetic framework used in this study was established previously based on lineage-specific consensus sequences of 167 random genes extracted from the RNAseq data [40], including sequences from *Fundulus heteroclitus* (GenBank accession number: JXMV00000000.1 [41]) that was used as an outgroup. Genes were concatenated and partitioned according to the most probably model of DNA substitution, as determined by *jModelTest* 2 v. 2.1.10 [42], and then used in maximum likelihood analyses using *RAxML* v. 8.2.9 [43]. *RAxML* analyses were conducted with the Rapid Bootstrap Algorithm with 1000 bootstrap replicates, and we used a thorough maximum likelihood (ML) search with each partition assigned its own GTR + Γ + I model. We recovered a robust phylogenetic tree consistent with previous poeciliid phylogenies [44–46]. Bootstrap support was mostly more than 99%. The only exceptions were *Gambusia eurystoma* and the two populations of *G. sexradiata* (bootstrap support 60%), which represent a complex of recently diverged species [47]. The best-scoring maximum likelihood tree was used in subsequent analyses.

### (d) Expression variance and evolution models

To identify convergent patterns in gene expression, we used the FPKM matrix (square-root-transformed) of the 11 390 filtered genes and the best-scoring maximum likelihood tree as inputs in expression variance and evolution (EVE) models, as implemented in the *twoThetaTest()* function from the EVEMODEL v. 0.0.0.9008 package [48] in *R* v. 4.6.0 [49]. EVE models implement an extended Ornstein–Uhlenbeck process that incorporates within-lineage expression variance to test for branch-specific shifts in gene expression by comparing likelihoods that an expression parameter (*θ_i_*) for a given gene is shared between two groups of lineages versus *θ_i_* for that gene being significantly different between the groups [50]. We designated branches associated with lineages from sulfidic habitats as one group and those associated with nonsulfidic lineages as another group and then contrasted *θ_i_* for each gene between these two groups. For each gene, we employed a likelihood ratio test (LRT*_θ_*), contrasting the null hypothesis (*θ_i_*^sulfidic^ = *θ_i_*^nonsulfidic^) to the alternative hypothesis (*θ_i_*^sulfidic^ ≠ *θ_i_*^nonsulfidic^) using a *χ*^2^ distribution to assess statistical significance [50]. To account for multiple testing, we calculated false-discovery-rate-(FDR)-adjusted *p*-values using the Benjamini–Hochberg procedure [51]. EVE models were run in this manner for each possible pair of sulfide spring lineages (total of 45 pairwise comparisons). Hence, the counts matrix and the best-scoring maximum likelihood tree were subset, such that each model included 10 nonsulfidic lineages in the background and 2 sulfidic lineages in the foreground. The number of genes with evidence for significant (FDR < 0.05) convergent expression shifts was then summed for each pair of sulfide spring lineages, providing a measure of the degree of convergence. Because each gene is evaluated against an explicit model-based null hypothesis within the EVE framework and significance is controlled using FDR correction across genes, the resulting number of significant shifts reflect deviations from an expected neutral model rather than raw expression similarity. Therefore, comparisons among lineage pairs are interpreted in a relative context, with all pairs evaluated under the same modelling framework and against a common set of background lineages.

### (e) Annotation of genes with signatures of convergence

To assess whether genes identified in our pairwise convergence analyses recovered biologically meaningful candidates in the context of known mechanisms of H_2_S adaptation, we conducted Gene Ontology (GO) enrichment analyses using GOrilla [52] on two sets of genes. First, we tested for enrichment of cellular components and biological functions among genes exhibiting convergent expression shifts in the majority of species pairs (i.e. greater than or equal to 23 of 45 pairs; supplementary material, table S2), using SwissProt IDs for the candidate gene set against a background consisting of all 11 390 genes included in the analysis. Second, we conducted the same analysis for genes exhibiting evidence of convergence in a minority of species pairs (i.e. less than 23 of 45 pairs; supplementary material, table S2). We predicted that convergent expression shifts would be enriched in mitochondrial pathways associated with H_2_S detoxification and energy metabolism, including enzymatic H_2_S oxidation in the sulfide : quinone oxidoreductase (SQR) pathway and components of oxidative phosphorylation and the respiratory chain involved in aerobic ATP production [18].

### (f) Phylogenetic and ecological distances

To test whether the degree of convergence in gene expression was associated with phylogenetic relatedness or ecological similarity between sulfide spring lineages, we first generated two predictor matrices. Phylogenetic relatedness was estimated by calculating phylogenetic distances between all lineage pairs based on the best-scoring maximum likelihood tree using the *cophenetic*() function from the STATS v. 4.6.0 package in R.

To calculate ecological similarity, we assembled two sets of environmental data. First, we measured aspects of water quality in all sulfide springs. Temperature, pH, specific conductivity and dissolved oxygen were measured using a Hydrolab Multisonde 4A (Hach Environmental, Loveland, CO, USA). Environmental H_2_S concentrations were measured with a methylene blue assay using a Hach DR1900 Portable Spectrophotometer (Hach Company, Loveland, CO, USA). Measurements and calibration of probes were conducted according to the manufacturers’ recommendations. Measurements for each site represent averages from at least three Hydrolab readings and two H_2_S samples, although estimates for most springs spanned repeated visits over multiple years. Second, we also downloaded 19 bioclimatic variables [53] based on the geographic location of each spring. Specifically, we used the *getData*() function from the RASTER v. 3.6.32 package [54] to download bioclim variables from the WorldClim [53] repository at a spatial resolution of 0.5 minutes of a degree. Bioclim variables included annual mean temperature (bio1), mean diurnal range (bio2), isothermality (bio3), temperature seasonality (bio4), maximum temperature of warmest month (bio5), minimum temperature of coldest month (bio6), temperature annual range (bio7), mean temperature of wettest quarter (bio8), mean temperature of driest quarter (bio9), mean temperature of warmest quarter (bio10), mean temperature of coldest quarter (bio11), annual precipitation (bio12), precipitation of wettest month (bio13), precipitation of driest month (bio14), precipitation seasonality (bio15), precipitation of wettest quarter (bio16), precipitation of driest quarter (bio17), precipitation of warmest quarter (bio18) and precipitation of coldest quarter (bio19; see supplementary material, table S3). To visualize variation in environmental conditions among springs, we conducted a principal component analysis with a correlation matrix on all 24 variables using the *prcomp*() function from the STATS v. 4.6.0 package in R. To obtain a metric of ecological dissimilarity for each pair of sulfide spring lineages, we calculated pairwise Euclidean distances in environmental PC space (first three dimensions) using the *dist*() function from the STATS v. 4.6.0 package. Calculating Euclidean distances based on environmental raw data yielded the same results, as measurements of environmental similarity in PC space (3 dimensions) and raw data (24 dimensions) were highly correlated (*r* = 0.992, *p* < 0.001).

It is important to note that phylogenetic distance and ecological distance were not significantly correlated in our dataset (*r* = −0.08, *p* = 0.60). Hence, more closely related lineages of sulfide spring fishes do not inhabit springs with more similar environmental conditions.

### (g) Hypothesis testing

To test hypotheses about the relationship between the degree of convergence, phylogenetic distance and ecological distance, we used a phylogenetic pairwise mixed modelling approach, which accounts for the non-independence inherent in species-pair observations [55,56]. Specifically, we modelled the degree of convergence between lineage pairs (square-root-transformed number of genes showing significant expression shifts) as a function of phylogenetic distance and ecological dissimilarity. Because each species appears in multiple pairwise comparisons, we incorporated phylogenetic covariance directly into the model using a species-level random effect structured by a phylogenetic covariance matrix derived from a phylogeny pruned to just include the sulfidic lineages. The phylogenetic covariance matrix was generated with the *vcv*() function from the APE v. 5.8.1 package [57].

We fit Bayesian multilevel models using the *brm*() function in the BRMS v. 2.23.0 package, which implements Hamiltonian Monte Carlo sampling via Stan [58]. The response variable was the square-root-transformed number of genes showing convergent expression shifts between lineage pairs. Fixed effects included phylogenetic distance, ecological dissimilarity and their interaction. To account for the non-independence inherent in pairwise data, we included random effects for both members of each species pair using a phylogenetically structured covariance matrix. This approach uses shared evolutionary history to account for covariance among species-level random effects, explicitly modelling phylogenetic non-independence and reciprocal dependence among observations within pairs.

Models were fit using four Hamiltonian Monte Carlo chains with 2000 iterations per chain (1000 warm-up and 1000 sampling iterations), yielding 4000 posterior draws per parameter. We used a conservative adapt_delta = 0.99 to reduce the risk of divergent transitions and improve sampling stability. Convergence was assessed using rank-normalized *R*-hat statistics and effective sample sizes for all parameters. Competing additive and interaction models were compared using Pareto-smoothed importance sampling leave-one-out (PSIS-LOO) cross-validation, with exact refitting (reloo) for observations exhibiting high influence. Posterior distributions were used to estimate effect sizes and associated 95% confidence intervals for all parameters, with inference based on the full posterior rather than point estimates.

## 3. Results

We analysed 118 gill transcriptomes from 10 lineages of sulfide spring poeciliids and 10 closely related lineages from adjacent nonsulfidic habitats. To quantify variation in the degree of convergence in gene expression between pairs of sulfide spring populations, we used expression levels of 11 390 genes across all lineages as dependent variables in EVE models, which were run separately for all pairwise combinations of sulfidic lineages. The number of convergent gene expression shifts (FDR < 0.05) identified through this approach ranged from 0 (between *L. sulphurophila* and multiple *Poecilia* species) to 412 (between *G. eurystoma* and *P. mexicana* Pich; figure 2A; supplementary material, table S2).

**Figure 2.**
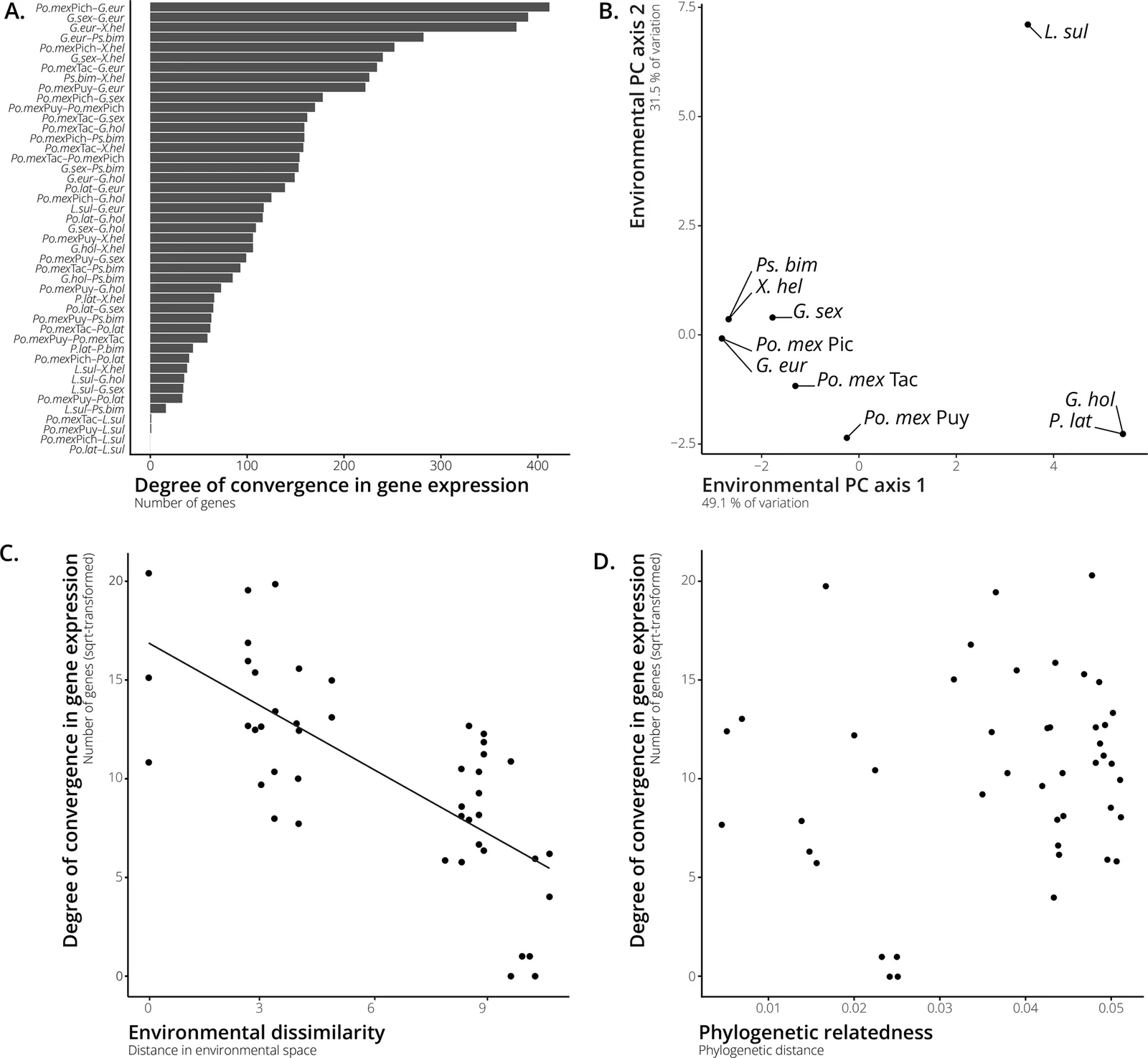
(A) Variation in the degree of convergence in gene expression among all pairs of lineages from sulfidic environments spanned over two orders of magnitude. (B) Visualization of environmental variation among different sulfide springs based on a principal component (PC) analysis. PC axis 1 was primarily correlated with pH, water temperature and H_2_S concentrations, as well as bioclimatic variables 1, 4, 6, 7, 9, 11, 12, 13, 14, 16, 17, 18 and 19 (see supplementary material, table S1, for details). Note that some springs harbour more than one lineage of H_2_S-adapted fish. (C) Variation in the degree of convergence between different sulfide spring lineages was strongly correlated with the environmental dissimilarity between their habitats (table 1). (D) Variation in the degree of convergence was not correlated with phylogenetic relatedness (table 1).

Some genes exhibited relatively consistent signatures of convergence across species pairs, appearing as significant in up to 38 of the 45 comparisons (supplementary material, table S2). Among the genes most frequently identified across multiple species pairs were several canonical H_2_S detoxification genes, including two copies of *ethe1* (persulfide dioxygenase), three copies of *mpst* (3-mercaptopyruvate sulfurtransferase) as well as *gsr* (glutathione reductase) and *sqrdl* (sulfide : quinone oxidoreductase) [59]. Consistent with theoretical predictions [18] and previous analyses [21–23,35,60], genes exhibiting convergent expression shifts in the majority (greater than or equal to 23) of comparisons were primarily active in mitochondria (supplementary material, figure S1) and associated with H_2_S metabolism and oxidation as well as sulfur amino acid processing (supplementary material, figure S2). Genes showing convergent expression shifts in a smaller subset of comparisons were likewise enriched for mitochondria and mitochondrial components (supplementary material, figure S3) and associated with biological functions known to be affected by H_2_S toxicity and detoxification, including aerobic metabolism, the function of the respiratory chain, glutathione metabolism, transsulfuration and mitochondrial translation (supplementary material, figure S4).

To test whether ecological similarity and phylogenetic relatedness predict the degree of convergence among lineages, we modelled the number of convergent expression shifts (square-root-transformed) as a function of pairwise environmental and phylogenetic distances, while accounting for shared evolutionary history using phylogenetic covariance and random effects for each lineage. To quantify ecological similarity among sulfide springs, we measured aspects of water quality *in situ* (temperature, pH, specific conductivity, dissolved oxygen and H_2_S concentration) and downloaded 19 geospatial variables describing climatic variation for each spring (supplementary material, table S3). Principal component (PC) analysis indicated that springs segregating along the first PC axis (figure 2B) differed in temperature, pH and H_2_S concentration, as well as bioclimatic variables associated with temperature and precipitation (correlations: |0.20| < *r* < |0.28|; supplementary material, table S4). In general, sulfide springs in Mexico (negative scores along PC axis 1) exhibited higher temperatures and H_2_S concentrations, lower pH, less seasonality and greater precipitation compared to springs in Florida and the Dominican Republic (positive scores along PC axis 1). Variation along the second PC axis was primarily driven by a single outlier, the sulfide spring from the Dominican Republic. Based on this multivariate environmental space, we calculated pairwise ecological distances among lineages and included them as predictors in the phylogenetic mixed model.

All models showed satisfactory convergence (*R*-hat ≈ 1.00) and high effective sample sizes (typically greater than 1000), with no indications of poor mixing. PSIS-LOO cross-validation indicated marginally higher expected log predictive density (ELPD) for the additive model (table 1). Consistent with the expectation that similar selective environments promote convergent shifts in gene expression, environmental distance was a strong negative predictor of convergence (*β* = −0.34, 95% CI: −0.51 to −0.17; table 1; figure 2C), indicating that lineages inhabiting more ecologically similar habitats exhibited a higher number of convergent gene expression shifts. By contrast, there was no evidence that more closely related lineages exhibited higher degrees of convergence than distantly related ones (*β* = 0.13, 95% CI: −0.22 to 0.44; table 1; figure 2D), indicating that evolutionary relatedness alone does not explain variation in convergence once ecological differences are considered.

**Table 1.** Results of an additive Gaussian phylogenetic mixed model fitted using Bayesian inference. Predictors were standardized (mean = 0, s.d. = 1). The model included phylogenetic covariance structures for both species identities (species 1 and species 2, as random effects). Posterior summaries were based on 4000 post-warmup draws from 4 chains. All *R*-hat values were approximately 1, and effective sample sizes were adequate. Note that the additive model shown here had marginally higher ELPD compared to the interactive model (supplementary material, table S5).

component	*β*	95% CI
intercept	−0.22 ± 0.69	−1.56 to 1.18
environmental distance	−0.34 ± 0.08	−0.51 to −0.17
phylogenetic distance	0.13 ± 0.16	−0.22 to 0.44
species 1 s.d. (intercept)	0.69 ± 0.26	0.32 to 1.33
species 2 s.d. (intercept)	1.05 ± 0.33	0.56 to 1.83
*σ* (residual)	0.31 ± 0.05	0.23 to 0.42

## 4. Discussion

Our phylogenetic comparative analyses of gene expression among sulfide spring fishes revealed starkly different patterns of convergent gene expression. The number of genes with evidence for convergent expression shifts varied over two orders of magnitude across lineage pairs. Genes with evidence for convergent expression shifts in the majority of comparisons were associated with H_2_S detoxification, including *ethe1*, *mpst* and *sqrdl*, all of which play critical roles in the enzymatic oxidation of H_2_S to nontoxic sulfur species [59]. Genes with evidence for convergent expression shifts in a minority of lineage pairs were associated with mitochondrial function and the respiratory chain, as expected given that H_2_S exerts its toxicity by reversibly binding to complex IV and disrupting aerobic ATP production in mitochondria [61]. Overall, our results are consistent with previous studies on a much smaller phylogenetic scale, which showed that different sulfide spring lineages of *Po. mexicana* consistently modulate genes associated with H_2_S detoxification [23], but only a subset of lineages also exhibits modifications to the respiratory chain that reduce the impact of H_2_S toxicity [20].

Most importantly, we found clear evidence that the degree of convergence in gene expression among sulfide spring lineages is related to the ecological similarity of their habitats. Lineage-specific idiosyncrasies are thus unlikely to be mere consequences of contingencies. Rather, fine-scale environmental variation, which is ignored when habitats are just classified based on the presence and absence of H_2_S [12], appears to influence the evolutionary trajectories of lineages in a consistent way. The importance of fine-scale environmental variation in shaping the degree of evolutionary convergence was also highlighted by a study of replicated pairs of three-spined stickleback (*Gasterosteus aculeatus*) that inhabit lake and stream habitats [16]. As in our study, the degree of convergence among population pairs was highly variable and related to differences in habitat structure, trophic resource use and parasite communities [16]. Our study highlights that the importance of fine-scale environmental variation that is often ignored in evolutionary studies does not only shape the degree of convergent evolution among closely related populations of the same species, where selection probably acts on shared pools of standing genetic variation, but it also does so at much broader phylogenetic scales [62].

If Gould was able to weigh in, he might caution us not to fall victim to the adaptationist fallacy [63]; after all, correlation does not imply causation. Future research needs to address how variation in complex organismal traits impacts the fitness of individuals in the context of the multifarious selective regimes found in sulfide springs. Unfortunately, this is not trivial empirically. While strong patterns of convergence provide tangible candidate traits to establish the relationships between form, function and fitness [21], the field of potential candidates is much wider when we consider traits that are modified in just one or a subset of lineages. This is a general problem in evolutionary analyses, and a major question moving forward is how we can be more explicit about the complexity of environments and organisms when studying evolutionary patterns and processes. Gaining a fuller picture about the function of organisms in their natural habitat will require us to move beyond reductionist approaches and consider that lineage-specific evolutionary patterns are not just noise but a consequence of deterministic forces and play a role in adaptation.

A major gap that remains unaddressed by our study is to what degree patterns of convergence in gene expression documented here are the product of evolutionary change in gene regulatory mechanisms as opposed to shared plastic responses to the environmental conditions that sulfide spring fishes encounter in their habitats. Studies in *Po. mexicana* have indicated that ancestral plasticity plays a minor role in shaping gene expression and other phenotypic differences between replicated sulfidic and nonsulfidic populations [35,64,65]. Instead, population differences in gene expression have a strong genetic basis and arise from changes in both constitutive expression and H_2_S-inducibility of genes involved in adaptation to sulfidic conditions [35], with regulatory divergence shaped by variation in promoters, enhancers, transcription factors and microRNAs [66–68]. The evidence for evolved changes in gene regulation at a much smaller phylogenetic scale—together with the high heritability of genome-wide mRNA expression patterns documented in other systems [69,70]—suggests that patterns of convergence across sulfide spring lineages are not merely a consequence of shared plastic responses but are at least in part shaped by adaptive evolution.

Our study also revealed that phylogenetic relatedness did not predict the degree of convergence in gene expression among different sulfide spring lineages. Closely related species are typically assumed to have a similar genetic architecture and may share patterns of genetic variation, leading to similar evolutionary responses when exposed to the same sources of selection [31,32,62]. One interpretation of our finding is that constraints associated with genetic variation and genomic architecture do not play a significant role in shaping responses to selection in sulfide springs. However, H_2_S targets ancient physiological pathways shared across metazoans. Its toxicity stems from interference with oxidative phosphorylation, a pathway highly conserved throughout the evolution of life [71,72], while detoxification occurs via the mitochondrial sulfide : quinone oxidoreductase pathway, which has prokaryotic origins and is likewise conserved across metazoans [73,74]. In most species, however, detoxification capacity is limited to endogenously produced H_2_S and rapidly overwhelmed by environmental exposure [75]. Consequently, adaptation to sulfide springs probably did not require the evolution of novel genes or functions, but rather modification of ancient pathways shared across lineages. Moreover, the well-defined biochemical and physiological effects of H_2_S may have constrained adaptive responses to a limited set of pathways, reducing the influence of among-lineage differences in genetic variation and genomic architecture on evolutionary outcomes [76]. This pattern may be particularly pronounced in gill tissue, which was the focus of our study, because gills represent the primary interface with the external environment and the major site of H_2_S exposure and detoxification [18]. Canonical H_2_S detoxification genes are strongly upregulated in gills [21–23,35], whereas interior organs such as the liver and brain generally exhibit much weaker transcriptional responses to sulfide exposure [77]. Consequently, gene expression in other tissues may show a stronger relationship with phylogenetic relatedness and lineage-specific history than observed here. In addition, insights from other study systems, where responses to selection are less constrained, will be critical to test the general role of phylogenetic distance in shaping the degree of convergent evolution in gene expression.

An additional source of variation that was not considered in our analyses is the age of adaptation to H_2_S-rich environments. Although ecological similarity explained substantial variation in the degree of convergent gene expression, lineages also differ in the duration over which selection has acted following colonization of sulfide springs [22,60]. Evolutionary responses to novel environments can change over time as populations accumulate genetic changes, potentially altering transcriptomic responses even under similar ecological conditions [78–80]. Consequently, time since colonization represents a potential source of variation in convergent gene expression that is distinct from ecological similarity and phylogenetic relatedness. Robust estimates of colonization history are currently unavailable for most sulfidic lineages, but incorporating this temporal dimension will be an important direction for future comparative analyses.

Finally, there appears to be a chasm between patterns of convergent evolution at a genomic level and emerging phenotypic traits. Convergence in phenotypes is common, but it is comparatively rare at the genomic level [11], unless constraints are substantial [81], selection acts on standing genetic variation [82], or adaptive alleles are introgressed across divergent lineages [83]. This chasm is also evident in the sulfide spring fishes studied here. Specifically, *Po. mexicana* (Pic), *Pseudoxiphophorus bimaculatus* and *X. hellerii*—which all inhabit the same sulfide spring—exhibit high levels of convergence in gene expression (figure 2A) and other traits [22]. Yet population genomic analyses revealed only scant evidence for convergence at a genomic level, and none of the outlier loci were associated with genomic regions containing genes associated with H_2_S toxicity and detoxification [22]. These contrasting results should not be surprising, and to some degree they reconcile the conflicting views Gould and Conway Morris held on the role of contingency and determinism in evolution. We have known since Kimura's introduction of the neutral theory that drift profoundly shapes genetic variation within and between species [84]. More so, redundancy in the genetic basis of quantitative traits involved in adaptation can cause idiosyncratic responses to selection that are in part influenced by chance [85,86]. Hence, genomic evolution largely reflects Gould's notion of the importance of contingency. But it is equally clear that contingent changes at a genomic level often give rise to deterministic evolutionary responses in phenotypes, even at the level of transcriptomes that follows just above the genome in the hierarchical organization of organisms. Importantly, cases of limited gene expression convergence among sulfide spring lineages are not simply unexplained noise. Rather, differences in the degree of transcriptomic convergence were associated with ecological differences among habitats, suggesting that lineage-specific outcomes can emerge through deterministic responses to fine-scale environmental variation. Our results indicate that deterministic outcomes are not restricted to traits that evolve in convergence across many taxa and respond to prominent sources of selection. Fine-scale environmental variation—which is subsumed into categorical habitat types when we employ reductionist approaches [12]—matters, because similar selective regimes beget similar evolutionary responses. Thus, the study of evolutionary repeatability has much to gain from multivariate approaches that reflect the complexity of organismal form and function and the multifarious nature of environmental gradients that exert selection on populations.

## Supplementary Material



## Data Availability

Data and code associated with all analyses are available on Zenodo [87] and Dryad [88]. Sequence data are available at the National Center for Biotechnology Information (NCBI) under Bio-Project accession numbers PRJNA473350 and PRJNA608180. Supplementary material is available online [89].

## References

[1] Blount ZD , Lenski RE, Losos JB. 2018 Contingency and determinism in evolution: replaying life's tape. Science 362, eaam5979. (doi:10.1126/science.aam5979) 30409860

[2] Gould SJ . 1990 Wonderful life: the burgess shale and the nature of history. New York, NY: W. W. Norton and Company.

[3] Conway Morris S . 2003 Life's solution: inevitable humans in a lonely universe. Cambridge, UK: Cambridge University Press. (doi:10.1017/CBO9780511535499)

[4] Beatty J . 2010Reconsidering the importance of chance variation. In Evolution: the extended synthesis (eds Pigliucci M, Müller GB), pp. 21–44. Cambridge, MA: MIT Press.

[5] Endler JA . 1986 Natural selection in the wild. Princeton, NJ: Princeton University Press.

[6] Schluter D . 2000 The ecology of adaptive radiation. Oxford, UK: Oxford University Press. (doi:10.1093/oso/9780198505235.001.0001)

[7] Losos JB . 2011 Convergence, adaptation, and constraint. Evolution 65, 1827–1840. (doi:10.1111/j.1558-5646.2011.01289.x) 21729041

[8] Stayton CT . 2015 What does convergent evolution mean? The interpretation of convergence and its implications in the search for limits to evolution. Interface Focus 5, 20150039. (doi:10.1098/rsfs.2015.0039) PMC4633856 26640646

[9] Ingram T , Mahler DL. 2013 SURFACE: detecting convergent evolution from comparative data by fitting Ornstein-Uhlenbeck models with stepwise Akaike Information Criterion. Methods Ecol. Evol. 4, 416–425. (doi:10.1111/2041-210X.12034)

[10] Stayton CT . 2015 The definition, recognition, and interpretation of convergent evolution, and two new measures for quantifying and assessing the significance of convergence. Evolution 69, 2140–2153. (doi:10.1111/evo.12729) 26177938

[11] Rosenblum EB , Parent CE, Brandt EE. 2014 The molecular basis of convergent evolution. Annu. Rev. Ecol. Evol. Syst. 45, 203–226. (doi:10.1146/annurev-ecolsys-120213-091851)

[12] Kaeuffer R , Peichel CL, Bolnick DI, Hendry AP. 2012 Parallel and nonparallel aspects of ecological, phenotypic, and genetic divergence across replicate population pairs of lake and stream stickleback. Evolution 66, 402–418. (doi:10.1111/j.1558-5646.2011.01440.x) PMC4499166 22276537

[13] Oomen RA , Kuparinen A, Hutchings JA. 2020 Consequences of single-locus and tightly linked genomic architectures for evolutionary responses to environmental change. J. Hered. 111, 319–332. (doi:10.1093/jhered/esaa020) PMC7423069 32620014

[14] Steiner CC , Rompler H, Boettger LM, Schoneberg T, Hoekstra HE. 2009 The genetic basis of phenotypic convergence in beach mice: similar pigment patterns but different genes. Mol. Biol. Evol. 26, 35–45. (doi:10.1093/molbev/msn218) 18832078

[15] Wainwright PC . 2005 Many-to-one mapping of form to function: a general principle in organismal design? Integr. Comp. Biol. 45, 256–262. (doi:10.1093/icb/45.2.256) 21676769

[16] Stuart YE *et al.* 2017 Contrasting effects of environment and genetics generate a continuum of parallel evolution. Nat. Ecol. Evol. 1, 0158. (doi:10.1038/s41559-017-0158) 28812631

[17] Tobler M , Kelley JL, Plath M, Riesch R. 2018 Extreme environments and the origins of biodiversity: adaptation and speciation in sulfide spring fishes. Mol. Ecol. 27, 843–859. (doi:10.1111/mec.14497) 29368386

[18] Tobler M , Passow CN, Greenway R, Kelley JL, Shaw JH. 2016 The evolutionary ecology of animals inhabiting hydrogen sulfide-rich environments. Annu. Rev. Ecol. Evol. Syst. 47, 239–262. (doi:10.1146/annurev-ecolsys-121415-032418)

[19] Barts N , Tobler M. 2023Adaptation to hydrogen sulfide: a case study in sulfide spring fishes. In Reference module in life sciences (ed. Alderman SL, Gillis TE), pp. 212–225. San Diego, CA: Academic Press.

[20] Pfenninger M *et al.* 2014 Parallel evolution of cox genes in H_2_S-tolerant fish as key adaptation to a toxic environment. Nat. Commun. 5, 3873. (doi:10.1038/ncomms4873) 24815812

[21] Greenway R *et al.* 2020 Convergent evolution of conserved mitochondrial pathways underlies repeated adaptation to extreme environments. Proc. Natl Acad. Sci. USA 117, 16424–16430. (doi:10.1073/pnas.2004223117) PMC7368198 32586956

[22] Greenway R *et al.* 2024 Integrative analyses of convergent adaptation in sympatric extremophile fishes. Curr. Biol. 34, 4968–4982.e7. (doi:10.1016/j.cub.2024.09.027) 39395416

[23] Kelley JL , Arias-Rodriguez L, Patacsil Martin D, Yee M-C, Bustamante CD, Tobler M. 2016 Mechanisms underlying adaptation to life in hydrogen sulfide-rich environments. Mol. Biol. Evol. 33, 1419–1434. (doi:10.1093/molbev/msw020) PMC4868117 26861137

[24] Riesch R , Plath M, Schlupp I, Tobler M, Brian Langerhans R. 2014 Colonization of toxic environments drives predictable life-history evolution in livebearing fishes (Poeciliidae). Ecol. Lett. 17, 65–71. (doi:10.1111/ele.12209) 24188245

[25] Tobler M , Hastings L. 2011 Convergent patterns of body shape differentiation in four different clades of poeciliid fishes inhabiting sulfide springs. Evol. Biol. 38, 412–421. (doi:10.1007/s11692-011-9129-4)

[26] Camarillo H , Arias Rodriguez L, Tobler M. 2020 Functional consequences of phenotypic variation between locally adapted populations: swimming performance and ventilation in extremophile fish. J. Evol. Biol. 33, 512–523. (doi:10.1111/jeb.13586) 31953965

[27] Tobler M , Palacios M, Chapman LJ, Mitrofanov I, Bierbach D, Plath M, Arias-Rodriguez L, García de León FJ, Mateos M. 2011 Evolution in extreme environments: replicated phenotypic differentiation in livebearing fish inhabiting sulfidic springs. Evolution 65, 2213–2228. (doi:10.1111/j.1558-5646.2011.01298.x) 21790570

[28] Tobler M , Scharnweber K, Greenway R, Passow CN, Arias‐Rodriguez L, García‐De‐León FJ. 2015 Convergent changes in the trophic ecology of extremophile fish along replicated environmental gradients. Freshw. Biol. 60, 768–780. (doi:10.1111/fwb.12530)

[29] Williams BL *et al.* 2026 Mechanisms underlying tolerance of severe hypoxia in sulfide spring fish. Comp. Biochem. Physiol. A Mol. Integr. Physiol. 319, 112038. (doi:10.1016/j.cbpa.2026.112038) 42320697

[30] Owens GL , Veen T, Moxley DR, Arias-Rodriguez L, Tobler M, Rennison DJ. 2021 Parallel shifts of visual sensitivity and body colouration in replicate populations of extremophile fish. bioRxiv. (doi:10.1101/2021.06.16.448734) 34784095

[31] Conte GL , Arnegard ME, Peichel CL, Schluter D. 2012 The probability of genetic parallelism and convergence in natural populations. Proc. R. Soc. B 279, 5039–5047. (doi:10.1098/rspb.2012.2146) PMC3497250 23075840

[32] Bohutínská M , Peichel CL. 2024 Divergence time shapes gene reuse during repeated adaptation. Trends Ecol. Evol. 39, 396–407. (doi:10.1016/j.tree.2023.11.007) 38155043

[33] Chaturvedi S , Gompert Z, Feder JL, Osborne OG, Muschick M, Riesch R, Soria-Carrasco V, Nosil P. 2022 Climatic similarity and genomic background shape the extent of parallel adaptation in Timema stick insects. Nat. Ecol. Evol. 6, 1952–1964. (doi:10.1038/s41559-022-01909-6) PMC7613875 36280782

[34] Evans DH , Claiborne JB. 2006 The physiology of fishes. Boca Raton, FL: Taylor & Francis.

[35] Passow CN , Henpita C, Shaw JH, Quackenbush CR, Warren WC, Schartl M, Arias‐Rodriguez L, Kelley JL, Tobler M. 2017 The roles of plasticity and evolutionary change in shaping gene expression variation in natural populations of extremophile fish. Mol. Ecol. 26, 6384–6399. (doi:10.1111/mec.14360) 28926156

[36] Krueger F . 2014 Trim galore! GitHub repository. https://github.com/felixkrueger/trimgalore.

[37] Warren WC *et al.* 2018 Clonal polymorphism and high heterozygosity in the celibate genome of the Amazon molly. Nat. Ecol. Evol. 2, 669–679. (doi:10.1038/s41559-018-0473-y) PMC5866774 29434351

[38] Li H , Durbin R. 2009 Fast and accurate short read alignment with Burrows-Wheeler transform. Bioinformatics 25, 1754–1760. (doi:10.1093/bioinformatics/btp324) PMC2705234 19451168

[39] Trapnell C , Williams BA, Pertea G, Mortazavi A, Kwan G, van Baren MJ, Salzberg SL, Wold BJ, Pachter L. 2010 Transcript assembly and quantification by RNA-seq reveals unannotated transcripts and isoform switching during cell differentiation. Nat. Biotechnol. 28, 511–515. (doi:10.1038/nbt.1621) PMC3146043 20436464

[40] Barts N , Greenway R, Passow CN, Arias-Rodriguez L, Kelley JL, Tobler M. 2018 Molecular evolution and expression of oxygen transport genes in livebearing fishes (Poeciliidae) from hydrogen sulfide rich springs. Genome 61, 273–286. (doi:10.1139/gen-2017-0051) 29227751

[41] Waits ER , Martinson J, Rinner B, Morris S, Proestou D, Champlin D, Nacci D. 2016 Genetic linkeage map and comparative genome analysis for the Atlantic killifish (*Fundulus heteroclitus*). Open J Genet. 06, 28–38. (doi:10.4236/ojgen.2016.61004)

[42] Darriba D , Taboada GL, Doallo R, Posada D. 2012 jModelTest 2: more models, new heuristics and parallel computing. Nat. Methods 9, 772. (doi:10.1038/nmeth.2109) PMC4594756 22847109

[43] Stamatakis A . 2014 RAxML version 8: a tool for phylogenetic analysis and post-analysis of large phylogenies. Bioinformatics 30, 1312–1313. (doi:10.1093/bioinformatics/btu033) PMC3998144 24451623

[44] Palacios M , Voelker G, Arias Rodriguez L, Mateos M, Tobler M. 2016 Phylogenetic analyses of the subgenus *Mollienesia* (*Poecilia*, Poeciliidae, Teleostei) reveal taxonomic inconsistencies, cryptic biodiversity, and spatio-temporal aspects of diversification in Middle America. Mol. Phylogenet. Evol. 103, 230–244. (doi:10.1016/j.ympev.2016.07.025) 27472959

[45] Hrbek T , Seckinger J, Meyer A. 2007 A phylogenetic and biogeographic perspective on the evolution of poeciliid fishes. Mol. Phylogenet. Evol. 43, 986–998. (doi:10.1016/j.ympev.2006.06.009) 17185005

[46] Reznick DN , Furness AI, Meredith RW, Springer MS. 2017 The origin and biogeographic diversification of fishes in the family Poeciliidae. PLoS One 12, e0172546. (doi:10.1371/journal.pone.0172546) PMC5344339 28278162

[47] Jourdan J , Krause ST, Lazar VM, Zimmer C, Sommer-Trembo C, Arias-Rodriguez L, Klaus S, Riesch R, Plath M. 2016 Shared and unique patterns of phenotypic diversification along a stream gradient in two congeneric species. Sci. Rep. 6, 38 971. (doi:10.1038/srep38971) PMC5159898 27982114

[48] Gillard GB *et al.* 2021 Comparative regulomics supports pervasive selection on gene dosage following whole genome duplication. Genome Biol. 22, 103. (doi:10.1186/s13059-021-02323-0) PMC8042706 33849620

[49] R Core Team. 2023 R: a language and environment for statistical computing. Vienna, Austria: R Foundation for Statistical Computing.

[50] Rohlfs RV , Nielsen R. 2015 Phylogenetic ANOVA: the expression variance and evolution (EVE) model for quantitative trait evolution. Syst. Biol. 64, 695–708. (doi:10.1093/sysbio/syv042) PMC4635652 26169525

[51] Benjamini Y , Hochberg Y. 1995 Controlling the false discovery rate: a practical and powerful approach to multiple testing. J. R. Stat. Soc. 57, 289–300. (doi:10.1111/j.2517-6161.1995.tb02031.x)

[52] Eden E , Navon R, Steinfeld I, Lipson D, Yakhini Z. 2009 GOrilla: a tool for discovery and visualization of enriched GO terms in ranked gene lists. BMC Bioinf. 10, 48. (doi:10.1186/1471-2105-10-48) PMC2644678 19192299

[53] Fick SE , Hijmans RJ. 2017 WorldClim 2: new 1-km spatial resolution climate surfaces for global land areas. Int. J. Climatol. 37, 4302–4315. (doi:10.1002/joc.5086)

[54] Hijmans RJ . 2021 raster: geographic data analysis and modelling. CRAN repository. See https://CRAN.R-project.org/package=raster.

[55] Hadfield JD , Nakagawa S. 2010 General quantitative genetic methods for comparative biology: phylogenies, taxonomies and multi-trait models for continuous and categorical characters. J. Evol. Biol. 23, 494–508. (doi:10.1111/j.1420-9101.2009.01915.x) 20070460

[56] Ives AR , Helmus MR. 2011 Generalized linear mixed models for phylogenetic analyses of community structure. Ecol. Monogr. 81, 511–525. (doi:10.1890/10-1264.1)

[57] Paradis E , Schliep K. 2019 ape 5.0: an environment for modern phylogenetics and evolutionary analyses in R. Bioinformatics 35, 526–528. (doi:10.1093/bioinformatics/bty633) 30016406

[58] Bürkner P-C . 2017 brms: an R package for Bayesian multilevel models using Stan. J. Stat. Softw. 80, 1–28. (doi:10.18637/jss.v080.i01)

[59] Olson KR . 2018 H_2_S and polysulfide metabolism: conventional and unconventional pathways. Biochem. Pharmacol. 149, 77–90. (doi:10.1016/j.bcp.2017.12.010) 29248597

[60] Brown AP , Arias-Rodriguez L, Yee M-C, Tobler M, Kelley JL. 2018 Concordant changes in gene expression and nucleotides underlie independent adaptation to hydrogen-sulfide-rich environments. Genome Biol. Evol. 10, 2867–2881. (doi:10.1093/gbe/evy198) PMC6225894 30215710

[61] Cooper CE , Brown GC. 2008 The inhibition of mitochondrial cytochrome oxidase by the gases carbon monoxide, nitric oxide, hydrogen cyanide and hydrogen sulfide: chemical mechanism and physiological significance. J. Bioenergy Biomembr. 40, 533–539. (doi:10.1007/s10863-008-9166-6) 18839291

[62] Boisseau RP , Bradler S, Emlen DJ. 2025 Divergence time and environmental similarity predict the strength of morphological convergence in stick and leaf insects. Proc. Natl Acad. Sci. USA 122, e2319485121. (doi:10.1073/pnas.2319485121) PMC11725862 39715436

[63] Gould SJ , Lewontin RC. 1979 The spandrels of San Marco and the Panglossian paradigm: a critique of the adaptationist programme. Proc. R. Soc. B 205, 581–598. (doi:10.1098/rspb.1979.0086) 42062

[64] Nobrega M , Greenway R, Passow CN, Arias Rodriguez L, Tobler M. 2024 Effects of plasticity and genetic divergence in phenotypic trait expression of sulfide spring fishes. Environ. Biol. Fishes 107, 611–629. (doi:10.1007/s10641-024-01555-w)

[65] Williams BL , Arias Rodriguez L, Schlupp I, Tobler M. 2026 Resilient by nature: managed rearing does not erode physiochemical tolerances of an extremophile fish. Biol. Lett. 22, 20 250 646. (doi:10.1098/rsbl.2025.0646) 41636208

[66] Kelley JL , Desvignes T, McGowan KL, Perez M, Rodriguez LA, Brown AP, Culumber Z, Tobler M. 2021 microRNA expression variation as a potential molecular mechanism contributing to adaptation to hydrogen sulfide. J. Evol. Biol. 34, 977–988. (doi:10.1111/jeb.13727) 33124163

[67] Perry BW , McGowan KL, Arias-Rodriguez L, Duttke SH, Tobler M, Kelley JL. 2024 Nascent transcription reveals regulatory changes in extremophile fishes inhabiting hydrogen sulfide-rich environments. Proc. Biol. Sci. 291, 20240412. (doi:10.1098/rspb.2024.0412) PMC11285508 38889788

[68] De-Kayne R , Perry BW, McGowan KL, Landers J, Arias-Rodriguez L, Greenway R, Rodríguez Peña CM, Tobler M, Kelley JL. 2024 Evolutionary rate shifts in coding and regulatory regions underpin repeated adaptation to sulfidic streams in poeciliid fishes. Genome Biol. Evol. 16, evae087. (doi:10.1093/gbe/evae087) PMC11126329 38788745

[69] Lloyd-Jones LR *et al.* 2017 The genetic architecture of gene expression in peripheral blood. Am. J. Hum. Genet. 100, 228–237. (doi:10.1016/j.ajhg.2016.12.008) PMC5294670 28065468

[70] Albert FW , Bloom JS, Siegel J, Day L, Kruglyak L. 2018 Genetics of trans-regulatory variation in gene expression. eLife 7, 35 471. (doi:10.7554/eLife.35471) PMC6072440 30014850

[71] Raanan H , Pike DH, Moore EK, Falkowski PG, Nanda V. 2018 Modular origins of biological electron transfer chains. Proc. Natl Acad. Sci. USA 115, 1280–1285. (doi:10.1073/pnas.1714225115) PMC5819401 29358375

[72] Saraste M . 1999 Oxidative phosphorylation at the fin de siècle. Science 283, 1488–1493. (doi:10.1126/science.283.5407.1488) 10066163

[73] Olson KR , Straub KD. 2016 The role of hydrogen sulfide in evolution and the evolution of hydrogen sulfide in metabolism and signalling. Physiology 31, 60–72. (doi:10.1152/physiol.00024.2015) 26674552

[74] Shahak Y , Hauska G. 2008Sulfide oxidation from cyanobacteria to humans: sulfide-quinone oxidoreductase (SQR). In Advances in photosynthesis and respiration (eds R Hell, C Dahl, D Knaff, T Leustek), pp. 319–335. Heidelberg: Springer.

[75] Bagarinao T . 1992 Sulfide as an environmental factor and toxicant: tolerance and adaptations in aquatic organisms. Aquat. Toxicol. 24, 21–62. (doi:10.1016/0166-445X(92)90015-F)

[76] Oakley TH . 2024 Building, maintaining, and (re-)deploying genetic toolkits during convergent evolution. Integr. Comp. Biol. 64, 1505–1512. (doi:10.1093/icb/icae114) 39025806

[77] Passow CN *et al.* 2017 Complexities of gene expression patterns in natural populations of an extremophile fish (*Poecilia mexicana*, Poeciliidae). Mol. Ecol. 26, 4211–4225. (doi:10.1111/mec.14198) PMC5731456 28598519

[78] Storz JF . 2016 Causes of molecular convergence and parallelism in protein evolution. Nat. Rev. Genet. 17, 239–250. (doi:10.1038/nrg.2016.11) PMC5482790 26972590

[79] Signor SA , Nuzhdin SV. 2018 The evolution of gene expression in cis and trans. Trends Genet. 34, 532–544. (doi:10.1016/j.tig.2018.03.007) PMC6094946 29680748

[80] Orr HA . 2005 The genetic theory of adaptation: a brief history. Nat. Rev. Genet. 6, 119–127. (doi:10.1038/nrg1523) 15716908

[81] Feldman CR , Brodie Jr ED, Brodie 3rd ED, Pfrender ME. 2012 Constraint shapes convergence in tetrodotoxin-resistant sodium channels of snakes. Proc. Natl Acad. Sci. USA 109, 4556–4561. (doi:10.1073/pnas.1113468109) PMC3311348 22392995

[82] Jones FC *et al.* 2012 The genomic basis of adaptive evolution in threespine sticklebacks. Nature 484, 55–61. (doi:10.1038/nature10944) PMC3322419 22481358

[83] Oziolor EM , Reid NM, Yair S, Lee KM, Guberman VerPloeg S, Bruns PC, Shaw JR, Whitehead A, Matson CW. 2019 Adaptive introgression enables evolutionary rescue from extreme environmental pollution. Science 364, 455–457. (doi:10.1126/science.aav4155) 31048485

[84] Kimura M . 1968 Evolutionary rate at the molecular level. Nature 217, 624–626. (doi:10.1038/217624a0) 5637732

[85] Barghi N *et al.* 2019 Genetic redundancy fuels polygenic adaptation in *Drosophila*. PLoS Biol. 17, e3000128. (doi:10.1371/journal.pbio.3000128) PMC6375663 30716062

[86] Barghi N , Hermisson J, Schlötterer C. 2020 Polygenic adaptation: a unifying framework to understand positive selection. Nat. Rev. Genet. 21, 769–781. (doi:10.1038/s41576-020-0250-z) 32601318

[87] Tobler M , Greenway R, Kelley JL. 2026 Data and scripts for: Ecology drives the degree of convergence in the gene expression of extremophile fishes. Zenodo. (doi:10.5281/zenodo.21920130) PMC13682929 42901522

[88] Tobler M , Greenway R, Kelley JL. 2026 Ecology drives the degree of convergence in the gene expression of extremophile fishes [Dataset]. Dryad. (doi:10.5061/dryad.8sf7m0d53) PMC13682929 42901522

[89] Tobler M , Greenway R, Kelley JL. 2026 Supplementary material from: Ecology drives the degree of convergence in the gene expression of extremophile fishes. Figshare. (doi:10.6084/m9.figshare.c.8690886) PMC13682929 42901522

