# Supplementary material for: Ecology drives the degree of convergence in the gene expression of extremophile fishes
Source: Proc Biol Sci. 2026 Oct 21;293(2081):20261416. doi: 10.1098/rspb.2026.1416 (PMC13682929; doi:10.1098/rspb.2026.1416)
Supplement: Supplementary file 1 [file rspb.2026.1416.zip › rspb.2026.1416/rspb20261416_si_001.pdf]

Online Supplementary Material

Ecology drives the degree of convergence in the gene expression of extremophile fishes

Proceedings of the Royal Society B; DOI: 10.1098/rspb.2026.1416

Data are available at Dryad; DOI: DOI: 10.5061/dryad.8sf7m0d53

Code is available at Zenodo; DOI: 10.5281/zenodo.21920130

Michael Tobler[1,2,3*], Ryan Greenway[4] & Joanna L. Kelley[5]

[1] Department of Biology, University of Missouri—St. Louis, St. Louis, MO 63121, USA

[2] Whitney R. Harris World Ecology Center, University of Missouri—St. Louis, St. Louis, MO 63121, USA

[3] WildCare Institute, Saint Louis Zoo, St. Louis, MO 63110, USA

[4] Max Planck Institute of Animal Behavior, 78464 Konstanz, Germany

[5] Department of Ecology and Evolutionary Biology, University of California Santa Cruz, Santa Cruz, CA 95064, USA

[*] Corresponding author: tobler@umsl.edu

Supplementary Figures

Figure S1. Results of a Cellular Component Gene Ontology enrichment analysis for genes exhibiting signatures of convergent expression shifts in the majority (≥23) of lineage pairs (also see Table S6). Note that this figure is provided as a separate file (FigureS1.png).

Figure S2. Results of a Biological Function Gene Ontology enrichment analysis for genes exhibiting signatures of convergent expression shifts in the majority (≥23) of lineage pairs (also see Table S6). Note that this figure is provided as a separate file (FigureS2.png).

Figure S3. Results of a Cellular Component Gene Ontology enrichment analysis for genes exhibiting signatures of convergent expression shifts in the minority (<23) of lineage pairs (also see Table S6). Note that this figure is provided as a separate file (FigureS3.png).

Figure S4. Results of a Biological Function Gene Ontology enrichment analysis for genes exhibiting signatures of convergent expression shifts in the minority (<23) of lineage pairs (also see Table S6). Note that this figure is provided as a separate file (FigureS4.png).

Supplementary Tables

Table S1. Locality information, latitude and longitude, as well as sample size (*N*) of all lineages included in this study.

Species	Locality	Lat/Long	*N*
Sulfidic lineages			
Poecilia mexicana (Puy)	La Lluvia springs, Rio Puyacatengo drainage, Tabasco, MX	17.464/-92.895	5
Poecilia mexicana (Tac)	El Azufre, Rio Tacotalpa drainage, Tabasco, MX	17.438/-92.775	5
Poecilia mexicana (Pic)	Baños del Azufre, Rio Pichucalco drainage, Tabasco, MX	17.552/-92.999	6
Poecilia latipinna	Green Springs, St. Johns River drainage, Florida, USA	28.863/-81.248	6
Limia sulphurophila	Balnearios La Zurza, Lago Enriquillo basin, Independencia, DR	18.398/-71.570	6
Gambusia sexradiata	Mogote del Puyacatengo, Rio Puyacatengo drainage, Tabasco, MX	17.582/-92.900	6
Gambusia eurystoma	Baños del Azufre, Rio Pichucalco drainage, Tabasco, MX	17.552/-92.999	6
Gambusia holbrooki	Green Springs, St. Johns River drainage, Florida, USA	28.863/-81.248	6
Pseudoxiphophorus bimaculatus	La Gloria springs, Rio Pichucalco drainage, Chiapas, MX	17.532/-93.015	6
Xiphophorus hellerii	La Gloria springs, Rio Pichucalco drainage, Chiapas, MX	17.532/-93.015	6
Nonsulfidic lineages			
Poecilia mexicana	Arroyo Bonita, Rio Tacotalpa drainage, Tabasco, MX	17.427/-92.752	6
Poecilia mexicana	Arroyo Rosita, Rio Pichucalco drainage, Chiapas, MX	17.485/-93.104	6
Poecilia mexicana	Rio Puyacatengo, Rio Puyacatengo drainage, Tabasco, MX	17.510/-92.914	6
Poecilia limantouri	Rio Garces, Rio Panuco drainage, Hidalgo, MX	20.940/-98.282	6
Poecilia latipinna	Mariner's Cove, Lake Monroe, St. Johns River drainage, Florida, USA	28.857/-81.239	6
Limia perugiae	Stream in Cabral, Rio Yaque del Sur drainage, Barahona, DR	18.246/-71.223	6
Gambusia sexradiata	Laguna Sitio Grande, Rio Ixtapangajoya drainage, Tabasco, MX	17.677/-92.997	6
Gambusia holbrooki	Mariner's Cove, Lake Monroe, St. Johns River drainage, Florida, USA	28.857/-81.239	6
Pseudoxiphophorus bimaculatus	Arroyo Pujil, Rio Ixtapangajoya drainage, Chiapas, MX	17.476/-92.986	6
Xiphophorus hellerii	Rio El Azufre, west branch, Rio Pichucalco drainage, Chiapas, MX	17.556/-93.008	6

Table S2. Summary of EVE results. The table includes a list of all 11,390 genes included in the analyses, their gene ID, SwissProt accession number and annotation, the number of models in which the gene exhibited a convergent expression shift, and the FDR-corrected *P*-value for each model. Note that this table is included as a separate spreadsheet (TableS2.csv).

Table S3. List of sulfide springs included in this study, including their geographic locations, latitude and longitude, the fish lineages inhabiting them, water quality parameters measured in situ, and bioclimatic variables download from WorldClim.

	**La Lluvia**	**El Azufre II**	**Baños del Azufre**	**La Gloria**	**Mogote del Puyacatengo**	**Green Springs**	**La Zurza**
Location	Chiapas, Mexico	Tabasco, Mexico	Tabasco, Mexico	Chiapas, Mexico	Tabasco, Mexico	Florida, USA	Dominican Republic
Lat/Long	17.464/-92.895	17.438/-92.775	17.552/-92.999	17.532/-93.015	17.582/-92.900	28.863/-81.248	18.398/-71.570
Lineage	*Po. mexicana* (Puy)	*Po. mexicana* (Tac)	*Po. mexicana* (Pic)<br>*G. eurystoma*	*Ps. bimaculatus*<br>*X. hellerii*	*G. sexradiata*	*Po. latipinna*<br>*G. holbrooki*	*L. sulphurophila*
DO [mg/l]	1.73	1.29	1.06	0.99	1.31	0.6	4.8
Temperature [°C]	25.7	27.9	31.2	29.4	27.2	26.3	26.2
pH	7.2	6.8	6.8	6.8	6.9	7.1	7.3
Specific conductivity [μScm^{-1}]	2285	3992	2697	2238	1931	2645	1257
H_2S [$\mu M/l$]	26.2	129.2	190.4	154.8	36.7	49.4	11.8
Bio1: Annual mean temperature	232	247	258	259	258	215	268
Bio2: Mean diurnal range	112	114	114	115	114	114	119
Bio3: Isothermality	66	66	65	64	65	46	77
Bio4: Temperature seasonality	1817	1851	1979	2030	1979	4733	1138
Bio5: Max. temperature of warmest month	316	335	348	351	348	328	342
Bio6: Min. temperature of coldest month	148	163	174	174	174	83	188
Bio7: Temperature annual range	168	172	174	177	174	245	154
Bio8: Mean temperature of wettest quarter	241	255	268	270	268	270	278
Bio9: Mean temperature of driest quarter	242	243	269	270	269	162	251
Bio10: Mean temperature of warmest quarter	250	266	278	280	278	270	281
Bio11: Mean temperature of coldest quarter	205	220	230	230	230	151	251
Bio12: Annual precipitation	3579	3189	3478	3637	3478	1306	842
Bio13: Precipitation of wettest month	518	493	535	553	535	184	136
Bio14: Precipitation of driest month	125	112	114	116	114	54	23

Bio15: Precipitation seasonality	43	45	45	46	45	45	54
Bio16: Precipitation of wettest quarter	1407	1292	1411	1491	1411	538	335
Bio17 Precipitation of driest quarter	439	397	386	386	386	176	72
Bio18 Precipitation of warmest quarter	689	641	800	847	800	531	266
Bio19 Precipitation of coldest quarter	687	583	701	739	701	206	72

Table S4. Results of the principal component analysis of environmental variables.

Variable	PC1	PC2	PC3
Dissolved oxygen	0.057	0.322	-0.223
Temperature	-0.217	0.004	0.299
pH	0.227	0.097	-0.276
Specific conductivity	-0.045	-0.235	0.053
H_2S concentration	-0.208	-0.053	0.287
Bio1: Annual mean temperature	-0.203	0.259	0.034
Bio2: Mean diurnal range	0.066	0.338	0.117
Bio3: Isothermality	-0.161	0.260	-0.224
Bio4: Temperature seasonality	0.204	-0.211	0.220
Bio5: Max. temperature of warmest month	-0.178	0.170	0.336
Bio6: Min. temperature of coldest month	-0.225	0.223	-0.085
Bio7: Temperature annual range	0.204	-0.206	0.231
Bio8: Mean temperature of wettest quarter	0.063	0.202	0.429
Bio9: Mean temperature of driest quarter	-0.261	0.154	-0.064
Bio10: Mean temperature of warmest quarter	-0.063	0.212	0.417
Bio11: Mean temperature of coldest quarter	-0.209	0.246	-0.079
Bio12: Annual precipitation	-0.273	-0.111	-0.067
Bio13: Precipitation of wettest month	-0.279	-0.095	-0.051
Bio14: Precipitation of driest month	-0.256	-0.162	-0.087
Bio15: Precipitation seasonality	0.084	0.344	0.027
Bio16: Precipitation of wettest quarter	-0.274	-0.110	-0.048
Bio17 Precipitation of driest quarter	-0.252	-0.164	-0.113
Bio18 Precipitation of warmest quarter	-0.241	-0.164	0.116
Bio19 Precipitation of coldest quarter	-0.273	-0.113	-0.035
Eigenvalue	3.432	2.751	1.833
Proportion of variance	0.491	0.315	0.140

Table S5. Results of an interactive Gaussian phylogenetic mixed model fitted using Bayesian inference. Predictors were standardized (mean = 0, SD = 1). The model included phylogenetic covariance structures for both species identities (species 1 and species 2, as random effects). Posterior summaries were based on 4,000 post-warmup draws from 4 chains. All R-hat values were ~1, and effective sample sizes were adequate. Note that leave-one-out cross-validation indicated no meaningful difference in predictive performance between the additive and interactive models (ΔELPD ± SE = −1.4 ± 3.2), and the confidence interval for the interaction term overlapped with zero, together suggesting that the interaction between environmental and phylogenetic distance did not significantly shape the degree of convergence observed in our dataset.

Component	β	95% CI
Intercept	–0.16 ± 0.59	–1.32 to 1.00
Environmental distance	–0.50 ± 0.10	–0.70 to –0.31
Phylogenetic distance	0.20 ± 0.18	–0.17 to 0.55
Environmental distance × phylogenetic distance	0.16 ± 0.10	–0.04 to 0.36
Species 1 SD (intercept)	0.62 ± 0.25	0.23 to 1.23
Species 2 SD (intercept)	0.79 ± 0.28	0.41 to 1.48
σ (residual)	0.38 ± 0.06	0.29 to 0.51

Table S6. Gene Ontology terms for genes exhibiting convergent shifts in gene expression in sulfide spring fishes. Provided are the GO term identification number, a description of each GO terms, P-value associated with the enrichment, false-discovery rate (q), the level of enrichment, the total number of genes in the target set (N), the total number of genes with a specific Go term in the reference set (B), the number of genes in the target set (n), and the number of genes in the intersection (b). We also provide a list of all genes with convergent expression shifts associated with each GO term. Note that this table is included as a separate spreadsheet (TableS6.csv).
